# Clinical and functional outcomes after laparoscopic IPOM repair: A comparison between hybrid biosynthetic and conventional meshes

**DOI:** 10.1007/s10029-026-03604-y

**Published:** 2026-03-25

**Authors:** Alessandro Verbo, Mattia Angelo Bez, Danilo Di Giorgio, Iacopo Verbo

**Affiliations:** 1 Catholic University of the Sacred Heart, Rome, Italy; 2 Mater Olbia Hospital, Olbia, Italy; 3 Sapienza University of Rome, Rome, Italy

**Keywords:** Ventral hernia, IPOM, Hybrid biosynthetic mesh, Quality of life, Hernia repair

## Abstract

**Background:**

Laparoscopic IPOM repair is widely used for ventral hernia treatment. Hybrid biosynthetic meshes have been developed to improve tissue integration and functional recovery; however, comparative clinical evidence remains limited.

**Methods:**

This was a single-center retrospective study conducted at a tertiary care institution between 2020 and 2025 including 95 consecutive patients undergoing laparoscopic IPOM repair. Forty-two patients received a hybrid biosynthetic mesh (SINECOR^®^) and 53 a conventional synthetic mesh. Demographic, clinical and surgical variables were analyzed, including defect size, mesh surface, operative time, complications, length of stay, functional and occupational recovery time, and quality of life (EuraHS-QoL). Hernia defects were classified as small (≤ 20 cm²), medium (21–100 cm²) and large (> 100 cm²). Mesh size was categorized as small (≤ 150 cm²), medium (151–400 cm²) and large (> 400 cm²). Delayed functional recovery was defined as occupational recovery > 30 days. Multivariable logistic regression was performed to identify independent predictors of delayed recovery; covariates included age > 65 years, BMI > 30 kg/m², defect size > 100 cm², mesh surface and mesh type (hybrid vs. conventional).

**Results:**

Groups were comparable in BMI (25.8 vs. 25.8 kg/m², *p* = 0.92), whereas patients in the hybrid group tended to be slightly older (63 vs. 58.5 years, *p* = 0.08). Follow-up was significantly longer in the control group (46.9 vs. 20.4 months, *p* < 0.001). No large defects (> 100 cm²) were observed in the hybrid mesh group, while they accounted for about one third of cases in the control group. Despite this imbalance, mean mesh surface did not differ significantly between groups (200.7 vs. 255.9 cm², *p* = 0.11). Hybrid mesh was associated with significantly shorter operative time (52.3 vs. 79.7 min, *p* < 0.001), reduced length of stay (2.0 vs. 2.8 days, *p* = 0.001), faster functional recovery (return to physical activity 10.5 vs. 13.5 days, *p* = 0.004; return to work 17.0 vs. 25.1 days, *p* < 0.001), and higher EuraHS-QoL scores (90.2 vs. 80.1, *p* < 0.001). Overall postoperative complication rates and seroma occurrence were comparable between groups. Recurrence was less frequent in the hybrid group (2.4% vs. 20.8%, *p* = 0.01) in the context of markedly shorter follow-up. On multivariable analysis, use of hybrid biosynthetic mesh remained the only independent predictor of faster functional recovery (OR 0.09; 95% CI 0.01–0.77; *p* = 0.028), with acceptable model calibration (Hosmer–Lemeshow *p* = 0.51; AUC = 0.73).

**Conclusions:**

In laparoscopic IPOM repair, hybrid biosynthetic mesh was associated with improved functional recovery and quality of life for small-to-medium defects, with a safety profile comparable to conventional meshes. Recurrence appeared lower with hybrid mesh, although interpretation is limited by shorter follow-up. Larger prospective studies are warranted to confirm these findings.

## Introduction

Ventral hernia repair is among the most frequently performed procedures in general surgery and its incidence is increasing due to population aging and the growing number of major abdominal operations. Despite technological progress and the widespread adoption of minimally invasive techniques, ventral hernia repair remains clinically challenging, particularly in terms of recurrence prevention, chronic pain reduction, and postoperative quality of life. The introduction of synthetic meshes has significantly reduced recurrence rates compared with primary suture repair, representing a major advance in abdominal wall surgery. However, conventional meshes may be associated with material stiffness, chronic inflammatory response, infection, adhesions, and persistent pain, potentially impairing functional outcomes and patient satisfaction. Recent advances in biomaterials have led to the development of biosynthetic and hybrid meshes designed to combine mechanical support with improved biocompatibility. In this context, a hybrid biosynthetic mesh composed of a permanent synthetic component combined with a bioabsorbable scaffold has been introduced to enhance tissue integration while maintaining structural strength. Despite increasing adoption, comparative clinical evidence remains limited and sometimes conflicting, particularly regarding long-term outcomes. Real-world data are therefore needed to better define the clinical impact of these devices. The aim of this study was to compare clinical, functional and quality-of-life outcomes after laparoscopic IPOM repair using a hybrid biosynthetic mesh versus conventional synthetic meshes, using a contemporary institutional database with systematic recording of perioperative and follow-up variables.

## Materials and methods

### Study design and population

A single-center retrospective observational study was conducted at a tertiary care institution between 2020 and 2025 in patients undergoing laparoscopic IPOM repair for ventral hernia. Ninety-five consecutive patients with complete clinical data and available follow-up were included: 42 treated with a hybrid biosynthetic mesh (SINECOR^®^, Gore, USA) and 53 with conventional synthetic meshes. Data were extracted from electronic medical records and operative logs and prospectively maintained institutional databases. Demographic, clinical and surgical variables were collected, including age, sex, body mass index (BMI), comorbidities, defect size, mesh surface, operative time, complications, length of hospital stay, time to functional and occupational recovery, and quality of life.

### Hernia and mesh classification

Hernia defects were classified based on preoperative imaging and intraoperative measurements. Defects were categorized according to surface area as small (≤ 20 cm²), medium (21–100 cm²), and large (> 100 cm²). Mesh size was categorized as small (≤ 150 cm²), medium (151–400 cm²), and large (> 400 cm²) according to the implanted prosthesis surface. In addition, hernias were retrospectively classified according to the European Hernia Society (EHS) classification for ventral hernias. Defect location was categorized as midline (M1–M4) or lateral (L1–L4), and defect width was classified as W1 (< 4 cm), W2 (4–10 cm), or W3 (> 10 cm), based on preoperative imaging and intraoperative measurements.

### Surgical technique

Primary fascial defect closure was performed whenever technically feasible, based on intraoperative assessment and surgeon judgment, taking into account defect size, tissue quality, and tension. This approach reflects the evolution of surgical practice during the study period and was not systematically applied in all cases. When performed, defect closure preceded mesh placement. When primary fascial closure was performed, it was achieved laparoscopically using an intracorporeal continuous suture prior to mesh placement; the decision to close the defect was based on intraoperative assessment of defect size, tissue quality, and tension.

### Perioperative management and follow-up

All procedures were performed by experienced abdominal wall surgeons using standardized laparoscopic IPOM techniques. Perioperative management followed institutional protocols, including antibiotic prophylaxis and thromboprophylaxis according to international guidelines. Follow-up included clinical and ultrasound evaluation at one month and subsequent outpatient visits, with additional imaging performed in case of symptoms or suspicion of recurrence. Time to functional recovery was defined as the interval from surgery to return to unrestricted daily physical activity as reported by the patient. Occupational recovery was defined as return to previous work activity. For the purposes of multivariable analysis, delayed functional recovery was defined as occupational recovery exceeding 30 days. Quality of life was assessed using the validated EuraHS-QoL questionnaire, administered during follow-up visits or via telephone interviews when appropriate.

### Outcomes and definitions

Postoperative pain and functional outcomes were primarily assessed using the validated EuraHS-QoL questionnaire, which evaluates pain, restriction of activities, and cosmetic discomfort. Formal postoperative pain scales such as visual analogue scale (VAS) scores were not systematically recorded. Mesh-related discomfort was therefore assessed indirectly through patient-reported outcomes captured within the EuraHS-QoL domains. Mesh fixation was performed according to surgeon preference, using resorbable tacks, transfascial sutures, or a combination of both. Fixation strategy was not standardized and was adapted to defect size, mesh surface, and intraoperative findings.

### Statistical analysis

Continuous variables were summarized as mean ± standard deviation or median and interquartile range, as appropriate, and compared using Student’s t-test or Mann–Whitney U test. Categorical variables were expressed as counts and percentages and compared using chi-square or Fisher’s exact test. Multivariable logistic regression was performed to identify independent factors associated with delayed functional recovery (> 30 days). Covariates included age > 65 years, BMI > 30 kg/m², defect size > 100 cm², mesh surface and mesh type (hybrid vs. conventional). Variables were selected based on clinical relevance and potential for confounding. Model calibration was assessed using the Hosmer–Lemeshow goodness-of-fit test, and discrimination using the area under the receiver operating characteristic curve (AUC). Statistical significance was set at *p* < 0.05. Given the sample size and event rate, the analysis was considered exploratory.

### Ethical approval

 According to national regulations, ethical approval was not required for this retrospective study. All data were anonymized.

## Results

### Baseline characteristics

 Baseline characteristics are reported in Table 3. Groups were comparable in BMI (25.8 ± 2.8 vs 25.8 ± 3.4 kg/m²; p = 0.92), while patients in the hybrid mesh group tended to be slightly older (63.0 ± 11.4 vs 58.5 ± 13.2 years; p = 0.08). Mean follow-up was significantly longer in the control group (46.9 ± 8.0 vs 20.4 ± 10.7 months; p < 0.001). No large defects (>100 cm²) were observed in the hybrid mesh group, whereas they accounted for approximately one third of cases in the control group (19/53, 35.8%). Small defects were more frequently treated with hybrid mesh. Despite this imbalance in defect size, mean mesh surface did not differ significantly between groups (200.7 ± 122.5 vs 255.9 ± 202.7 cm²; p = 0.11), with a similar distribution of small, medium and large prostheses.

### Operative and postoperative outcomes

 Hybrid mesh implantation was associated with significantly shorter operative time compared with conventional meshes (52.3 ± 21.3 vs 79.7 ± 47.5 minutes; p < 0.001). Length of hospital stay was also reduced in the hybrid group (2.0 ± 0.5 vs 2.8 ± 1.6 days; p = 0.001). Postoperative complications occurred in 5/42 patients (11.9%) in the hybrid group and 8/53 (15.1%) in the control group (p = 0.88). Seroma was the most common event in both groups, with similar incidence. No signal of increased major complications was observed with hybrid mesh.

### Functional recovery and quality of life

 Functional outcomes favored the hybrid biosynthetic mesh. Time to resumption of physical activity was shorter in the hybrid group (10.5 ± 3.7 vs 13.5 ± 6.1 days; p = 0.004). Occupational recovery was also significantly faster (17.0 ± 5.5 vs 25.1 ± 13.3 days; p < 0.001). Delayed functional recovery (>30 days) occurred in 1/42 patients (2.4%) in the hybrid group and 9/53 (17.0%) in the control group. Quality of life, as measured by the EuraHS-QoL score, was higher in the hybrid mesh group (90.2 ± 8.2 vs 80.1 ± 15.6; p < 0.001), reflecting better patient-reported outcomes in terms of symptoms, daily activities and cosmetic satisfaction.

### Recurrence

 During follow-up, hernia recurrence was documented in 1/42 patients (2.4%) in the hybrid mesh group and 11/53 (20.8%) in the control group (p = 0.01). Given the significantly longer follow-up in the conventional mesh group and the time-dependent nature of ventral hernia recurrence, this difference should be interpreted with caution.

### Multivariable analysis

 Multivariable logistic regression for delayed functional recovery (>30 days) is summarized in Table 1. After adjustment for age >65 years, BMI >30 kg/m², defect size>100 cm² and mesh surface, the use of hybrid biosynthetic mesh remained the only independent predictor of faster functional recovery (OR 0.09; 95% CI 0.01–0.77; p = 0.028). Other covariates were not significantly associated with delayed recovery. The model showed acceptable calibration (Hosmer–Lemeshow p = 0.51) and fair discrimination (AUC 0.73), despite the limited number of events. Subgroup analysis according to defect size and patient characteristics (Table 2) suggested that the greatest benefit of hybrid mesh in terms of recovery and quality of life was observed in medium-sized defects, whereas outcomes were more comparable in very small and more complex anatomical scenarios. Table 3.

**Table 1:** Multivariable logistic regression analysis for predictors of delayed functional recovery (> 30 days)

Predictor	**Odds Ratio (OR)**	**95% CI**	**p-value**
Age >65 years	1.42	0.28 – 7.10	0.67
BMI >30 kg/m²	1.18	0.18 – 7.67	0.86
Defect size >100 cm²	2.01	0.40 – 10.1	0.39
Mesh surface (per 100 cm²)	1.04	0.97 – 1.12	0.25
Hybrid mesh (vs conventional)	**0.09**	**0.01 – 0.77**	**0.028**

**Table 2:** Subgroup analysis of functional recovery according to defect size and patient characteristics

Subgroup	**Hybrid mesh (mean ± SD)**	**Conventional mesh (mean ± SD)**	**p-value**
Small defects (≤20 cm²)	Return to work: 15.2 ± 4.9 d	20.8 ± 10.2 d	0.03
Medium defects (21–100 cm²)	Return to work: 17.9 ± 5.8 d	27.5 ± 14.1 d	<0.001
Large defects (>100 cm²)	*n = 0*	29.4 ± 11.6 d	—
Age >65 years	18.2 ± 6.0 d	25.9 ± 13.7 d	0.01
BMI >30 kg/m²	19.0 ± 5.5 d	26.4 ± 14.0 d	0.04

**Table 3:** Demographic and clinical characteristics of the study population

Variable	**Hybrid mesh (n=42)**	**Conventional mesh (n=53)**	**p-value**
Age (years)	63.0 ± 11.4	58.5 ± 13.2	0.08
Sex (M/F)	19/23	22/31	0.83
BMI (kg/m²)	25.8 ± 2.8	25.8 ± 3.4	0.92
Defect size (cm²)	33.7 ± 28.2	73.6 ± 85.9	<0.001
• Small (≤20 cm²)	19 (45.2%)	16 (30.2%)	0.14
• Medium (21–100 cm²)	23 (54.8%)	18 (34.0%)	0.04
• Large (>100 cm²)	0 (0%)	19 (35.8%)	<0.001
Mesh surface (cm²)	200.7 ± 122.5	255.9 ± 202.7	0.11
• Small (≤150 cm²)	22 (52.4%)	18 (34.0%)	0.07
• Medium (151–400 cm²)	19 (45.2%)	25 (47.2%)	0.85
• Large (>400 cm²)	1 (2.4%)	10 (18.8%)	0.02
Operative time (min)	52.3 ± 21.3	79.7 ± 47.5	<0.001
Hospital stay (days)	2.0 ± 0.5	2.8 ± 1.6	0.001
Complications (overall)	5 (11.9%)	8 (15.1%)	0.88
Seroma	3 (7.1%)	4 (7.5%)	0.94
Return to physical activity (days)	10.5 ± 3.7	13.5 ± 6.1	0.004
Return to work (days)	17.0 ± 5.5	25.1 ± 13.3	<0.001
Delayed recovery >30 days	1 (2.4%)	9 (17.0%)	0.02
EuraHS-QoL score	90.2 ± 8.2	80.1 ± 15.6	<0.001
Recurrence	1 (2.4%)	11 (20.8%)	0.01
>Follow-up (months)	20.4 ± 10.7	46.9 ± 8.0	<0.001

## Discussion

 In this single-center retrospective analysis of laparoscopic IPOM repair, hybrid biosynthetic mesh was associated with superior functional outcomes, including shorter operative time and hospital stay, faster recovery, and improved quality of life, compared with conventional meshes. These findings are consistent with previous evidence suggesting that biosynthetic and hybrid meshes may be associated with reduced chronic pain and greater patient satisfaction compared with permanent synthetic materials [1–3]. Given the increasing emphasis on patient-reported outcomes, the observed improvement in EuraHS-QoL scores is clinically relevant. Quality of life has been demonstrated to represent a fundamental determinant of perceived surgical success following ventral hernia repair [2]. The shorter operative time observed in the hybrid mesh group may plausibly relate to improved handling characteristics and material conformability, as reported in previous clinical experiences with biosynthetic and hybrid meshes [4, 5]. Although operative time is not a direct clinical endpoint, it may influence anesthetic exposure and postoperative recovery, particularly in elderly or comorbid patients. The numerically and statistically lower recurrence rate observed in the hybrid group in our cohort should nonetheless be interpreted cautiously, given the longer follow-up in the control group and the time-dependent nature of ventral hernia recurrence [6]. It is conceivable that differences in recurrence may attenuate over extended follow-up, and longer-term data are needed before drawing firm conclusions. Differences observed between the hybrid and conventional mesh groups should be interpreted in light of relevant baseline imbalances. In particular, the hybrid mesh group included fewer patients, smaller defect sizes, and a substantially shorter follow-up duration compared with the control group. These factors may have influenced operative time, length of hospital stay, and functional recovery, independently of mesh material. Similarly, recurrence rates are inherently time-dependent and may be underestimated in the hybrid group due to shorter surveillance. For these reasons, comparative outcomes should be considered exploratory and hypothesis-generating rather than conclusive. Multivariable analysis identified hybrid mesh as the only independent predictor of faster functional recovery, supporting a potential material–tissue interaction effect on early postoperative outcomes. This observation is consistent with experimental evidence suggesting that mesh integration and modulation of the local inflammatory response play a key role in long-term success following abdominal wall reconstruction [3, 9]. Subgroup analysis demonstrated a more pronounced benefit in patients with medium-sized defects, suggesting that hybrid meshes may provide the greatest advantage in anatomically intermediate scenarios, where an optimal balance between mechanical support and biocompatibility is required [7, 10]. Safety outcomes were comparable between groups, in agreement with reported data indicating no significant differences in major complication rates between synthetic, biosynthetic and hybrid meshes when properly selected [1, 4, 8, 11]. Importantly, the use of a hybrid biosynthetic mesh in our series did not translate into an increase in seroma, infection or other clinically relevant adverse events. Several limitations should be acknowledged. First, the retrospective design and limited sample size reduce the generalizability of these findings and may have introduced unmeasured confounding. Second, heterogeneity in follow-up duration, with substantially longer surveillance in the control group, limits definitive evaluation of recurrence rates. Third, detailed comorbidity burden and anesthesiologic risk scores were not systematically available in the database, precluding their full incorporation into multivariable modeling. Finally, the absence of large defects in the hybrid group reflects a degree of surgeon-driven selection and limits extrapolation of our findings to more complex hernia repairs. Overall, although our data suggest that hybrid biosynthetic mesh represents a valuable alternative to conventional materials for small-to-medium defects, larger prospective randomized trials with standardized follow-up and stratification by defect complexity are required to define optimal indications and to clarify long-term recurrence patterns.

### Limitations

 Because primary fascial defect closure was not mandated by protocol and evolved over the study period, it was not uniformly applied across patients. This variability may have influenced postoperative outcomes such as recurrence, seroma formation, and postoperative pain, and should be considered when interpreting the results.

### Conclusions

In laparoscopic IPOM repair, hybrid biosynthetic mesh was associated with faster functional recovery and improved postoperative quality of life for small-to-medium defects, with no increase in complications compared with conventional meshes. Recurrence appeared lower in the hybrid group, but this finding must be interpreted in light of shorter follow-up and the exploratory nature of the study. These results support the use of hybrid biosynthetic mesh as an effective and safe option in selected patients and warrant confirmation in prospective randomized studies with standardized follow-up.

## Data Availability

The datasets generated and analyzed during the current study are available from the corresponding author upon reasonable request.
